# Seizure as an Atypical Presentation of Myasthenia Gravis: A Rare but Critical Diagnostic Challenge

**DOI:** 10.1002/ccr3.72188

**Published:** 2026-03-05

**Authors:** Huda Faisal, Chanchan, Karan Chaman Lal, Zahabia Adnan, Shamyl Zehra, MD. Faisal Ahmed

**Affiliations:** ^1^ Department of Medicine Peoples University of Medical & Health Sciences Nawabshah Sindh Pakistan; ^2^ Department of Medicine Liaquat University of Medical & Health Sciences Jamshoro Sindh Pakistan; ^3^ Department of Medicine Jinnah Sindh Medical University Karachi Sindh Pakistan; ^4^ Department of Health Science and Informatics Bangladesh Institute of Innovative Health Research Dhaka Bangladesh

**Keywords:** acetylcholine receptor antibody (AchR), epilepsy, IgG autoantibodies, myasthenia gravis, seizures

## Abstract

A 27‐year‐old male patient with a known case of epilepsy and also recently diagnosed with myasthenia gravis presented with dysphagia, respiratory distress, and seizures. Despite timely intervention, he progressed into septic shock during a myasthenic crisis and succumbed to cardiopulmonary arrest. The underlying autoimmune nature of this condition shows a clinically significant and rare overlap between myasthenia gravis and epilepsy. This case emphasizes the importance of early detection and careful drug selection or management in neuromuscular and neurological disorders.

## Key Clinical Message

The unusual co‐occurrence of Myasthenia Gravis and epilepsy highlighted the importance of considering multiple possible diagnoses to ensure patient survival through insightful surveillance, interdisciplinary management, and careful drug choice. The identification of autoimmune overlap with neurological diseases not only improves diagnosis and treatment but also encourages researchers to investigate further.

## 1 Introduction

Myasthenia Gravis is an autoimmune disease in which the IgG autoantibodies are predominantly formed in 74%–88% of cases, which destroy the nicotinic receptor; therefore, acetylcholine is unable to perform its activity, which can significantly lead to muscle weakness and fatigue due to loss of contractility [1, 2]. Globally, 40–80 per million cases of Myasthenia Gravis prevail, and the annual incidence of the disease is 4–12 per million cases, depicting its rarity [3]. In the United States, the burden of Myasthenia Gravis has increased to about 20 per 100,000 cases [4]. Wide disparities were noted among gender and age, with a female‐to‐male ratio of 3:1, with a high spectrum of disease seen below 40 years of age. However, between the ages of 40–50 years, the ratio seems to be equal and more frequent in males [5].

Moreover, epileptic seizures are abnormal, involuntary, and rapid jerky movements due to neuronal excitation or inhibition within the brain. An imbalance between the inhibitory neurotransmitter GABA and the excitatory neurotransmitter Glutamate results in abnormal movements leading to the development of seizures. This complex neurological ailment affects around 0.5%–1.0% of the population globally [6]. Approximately one‐third of seizures are due to genetic abnormalities involving ion channels, and around a quarter of seizures are due to structural lesions [7].

The coexistence between myasthenia gravis and seizures is relatively uncommon, but may occur primarily due to autoimmune disease [8]. Moreover, in Myasthenia Gravis, due to respiratory muscle weakness, a patient can develop hypoxia, hypoventilation, and sleep apnea as a complication, leading to an abnormal EEG pattern and thus neurological impairment, particularly episodes of seizures occurring [6, 9]. Quite limited, but existing literature reported that some medications may also influence these conditions. To document an unusual coexistence between Myasthenia Gravis and seizures, a rare case with such findings was presented in a routine clinical setup, with special emphasis on autoimmune associations, diagnostic difficulties, and considerations in management.

## 2 Case History/ Examination

A 27‐year‐old male, a known case of epilepsy who had poor compliance with medications, was admitted to the emergency department. He had complaints of dysphagia of duration of 2 months, shortness of breathing (SOB) of 4 days, and an untreated fever of 2 days. His difficulty in swallowing had been progressive, which had initially affected solids but later progressed to liquids. The patient also reported associated regurgitation of the swallowed liquids from the mouth and nose. He had SOB that had progressively become worse over 1 week with no history of chills, sweats, cough, orthopnea, or paroxysmal nocturnal dyspnea. He never had aspiration phenomena, chest pain, hay sputum of blood clots, or SOB on cold nights, although he had experienced several episodes of undocumented fever in recent weeks.

He had known epilepsy, which was easily managed on levetiracetam but was poorly controlled as a result of noncompliance. He was found to have Myasthenia Gravis based on positive serum acetylcholine receptor antibodies and abnormal repetitive nerve stimulation. However, he was on 60 mg of pyridostigmine (Mestinon) once daily. He had no history of substance abuse, and past surgical or family histories were unremarkable.

Diagnostic and therapeutic modalities would not have been feasible in the facility due to resource constraints. These findings are limited by a lack of on‐site neurophysiology and unreliable radiography (no available EEG or chest imaging). Furthermore, the patient volume and turnover were extremely high during this time period, further constraining access to the already limited diagnostic capacity. Treatment plans and clinical judgments, therefore, had to be largely based on bedside examination and readily accessible laboratory information.

## 3 Methods

### 3.1 Differential Diagnosis and Investigations

On general physical examination, the patient was ill but negative for pallor, jaundice, cyanosis, edema, and clubbing; his vitals were: blood pressure 144/87 mmHg, pulse rate 124 beats/min, respiratory rate of 18 breaths/min, oxygen saturation of 96% on room air, and random blood sugar of 386 mg/dL. On neurological examination, GCS was 4/15 (E1V0M3), bilaterally constricted pupils sluggishly reactive to light, and normal muscle tone. Respiratory examination showed bilaterally normal vesicular breath sounds, whereas cardiovascular examination revealed audible S1 and S2 without any added murmurs or gallops. Abdominal examination showed a soft, non‐tender abdomen with a palpable liver.

### 3.2 Neuroimaging

**FIGURE 1:**
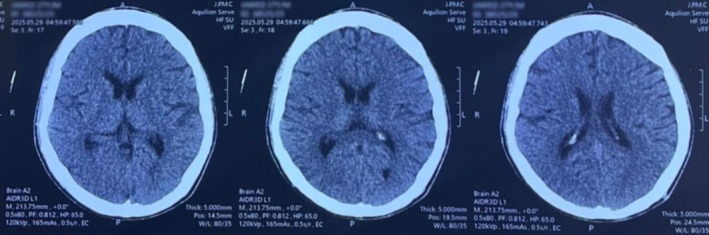
The non‐contrast computed tomography scan of the brain demonstrated a normal size and contour of the ventricles, with a distinct differentiation between gray and white matter. There were no indications of intracranial hemorrhage, infarction, mass lesions, or midline shift. Additionally, the basal cisterns, cortical sulci, and bony calvarium were observed to be normal and intact.

**FIGURE 2:**
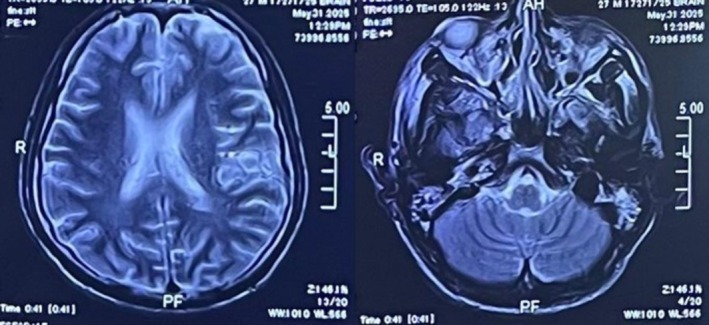
The contrast‐enhanced magnetic resonance imaging of the brain revealed normal parenchymal architecture and consistent signal intensity across all sequences. No abnormal contrast enhancement, demyelinating plaques, infarcts, or space‐occupying lesions were identified. The ventricular system and midline structures were well‐aligned, and on further evaluation, the posterior fossa, brainstem, and cerebellum yielded no abnormalities, confirming the absence of meningeal enhancement or paranasal sinus pathology.

A non‐contrast CT scan of the brain revealed normal ventricular size and configuration with maintained gray–white matter differentiation. No intracranial hemorrhage, infarction, mass lesion, or midline shift was seen. This excludes any acute intracranial abnormality, effectively ruling out structural causes for seizure, shown in Figure 1. A contrast‐enhanced MRI brain, as shown in Figure 2, showed normal cerebral parenchyma, no abnormal enhancement, demyelinating disease, infarcts, or space‐occupying lesions, excluding structural or demyelinating causes of seizures.

### 3.3 Serology

Serum acetylcholine receptor antibody (AchR) was detected positively, and Myasthenia Gravis was diagnosed in Table 1, ruling out other systemic illnesses like systemic lupus erythematosus (SLE), Rheumatoid arthritis (RA), Connective tissue diseases, and others. However, serum electrolytes, Liver function tests, and coagulation profile were normal, eliminating metabolic, hepatic etiology of altered sensorium or seizures and ruling out bleeding tendency, respectively. Complete blood count revealed mild normocytic anemia but no leukocytosis or signs of active infection. The blood cultures were negative, and the urinalysis was normal.

**TABLE 1:** Laboratory investigation.

Test	Lab value	Result	Normal range
Serum anti‐musk antibodies	< 0.1 U/mL	Normal	< 0.4 U/mL
Serum acetylcholine receptor antibody	35.20 nmol/L	Positive	Negative: < 0.40 nmol/L Intermediate: 0.40–0.49 nmol/L Positive: > 0.5 nmol/L

*Note:* Serum AChR and MuSK Antibody profile presents a high titer of antibodies against acetylcholine receptor (35.20 nmol/L), strongly suggestive of autoimmune Myasthenia Gravis, as the MuSK antibody has a negative value (< 0.1 U/mL), meaning absence of the MuSK subtype form of Myasthenia Gravis. ANA, AMA, and ASMA profiles are all negative, which practically excludes any systemic autoimmune or connective tissue diseases (systemic lupus erythematosus, autoimmune hepatitis or primary biliary cirrhosis). Together, these results support the diagnosis of AChR antibody–positive generalized Myasthenia Gravis without evidence for overlapping autoimmunity.

### 3.4 Treatment

In the emergency room, the patient was managed with intravenous Levetiracetam 1.2 g in normal saline 100 mL, Ceftriaxone 2 g, Haloperidol 5 mg, and Solucortef 250 mg. Face mask oxygen therapy at a rate of 10 L per minute was started. Subsequently, he was then transferred to the ward, where he kept on ventilatory support, insulin infusion for hyperglycemia, and intravenous Dormicum, Ceftriaxone, and Meropenem.

## 4 Conclusion and Results

Despite aggressive medical and supportive management, the patient's respiratory condition worsened rapidly, leading to septic shock secondary to myasthenic crisis, with eventual cardiopulmonary arrest. All resuscitation attempts failed, and the patient expired.

## 5 Discussion

The unusual coexistence of Myasthenia Gravis and Epilepsy is sparsely documented in the existing literature. A few hypotheses highlight the pathophysiological correlation between the two. Firstly, the molecular similarity between immune factors may cause a person to develop a tendency toward autoimmunity, making them more susceptible to both conditions. This shared genetic susceptibility, including similar HLA Class II variations, makes a patient prone to develop more than one autoimmune condition, which ultimately increases the vulnerability to develop CNS autoimmunity too. Secondly, in MG, levels of certain inflammatory cytokines, such as IL‐6 and TNF‐α, increase, which makes the neurons easily excitable, lowering the threshold for seizures to occur. These high levels of cytokines also transiently impair the blood–brain barrier and increase permeability to circulating autoantibodies, which may provoke epileptic seizures. Additionally, the use of certain medications to treat Myasthenia Gravis, such as fluoroquinolones, macrolides, and high‐dose steroids, can actually aggravate the risk of seizure development, as they interfere with the mechanism of neurotransmitters like GABA [10]. Similarly, many reported cases show that the use of antiepileptic drugs like phenytoin can increase the risk of acute respiratory failure [11]. Moreover, the association of thymic abnormalities in MG results in T‐cells escaping into circulation and later attacking the brain‐specific proteins, causing neurological impairment.

A careful diagnostic assessment is necessary to handle such a patient, as both Central Nervous System (CNS) and Peripheral Nervous System (PNS) symptoms were involved. Myasthenia Gravis was confirmed by the detection of AChR antibodies and by the Repetitive Nerve Stimulation (RNS) findings; secondary outcomes, which show structural and metabolic causes of seizures, were excluded from laboratory findings. This is because it highlights the importance of rule out phenomena and comprehensive evaluation for autoimmune overlap syndromes.

Early identification of respiratory insufficiency in Myasthenia Gravis patients may preclude hypoxia‐associated neurological sequelae [12]. Optimal management of Myasthenia Gravis with seizures requires an interdisciplinary plan involving intensive care, immunology, and neurology. The choice of medication must avoid drugs that aggravate one or both conditions; Levetiracetam is the drug of choice because of its few neuromuscular side effects. Ventilatory monitoring at all times is important because hypoxia can induce seizures. Immunotherapy needs to be employed carefully because corticosteroids can worsen Myasthenia Gravis and decrease seizure threshold. Adherence to antiepileptic treatment is essential to avoid recurrence and enhance general patient stability.

This case points out the unusual coexistence of Myasthenia Gravis with epilepsy, emphasizing the requirement for close monitoring and an interdisciplinary management strategy. Optimal drug selection, prompt identification of respiratory distress, and treatment adherence are important. Elucidation of common autoimmune mechanisms may promote better insight and permit more accurate, targeted therapeutic interventions.

## Author Contributions

**Huda Faisal:** conceptualization, formal analysis, investigation, writing – original draft, writing – review and editing. **Chanchan:** writing – original draft, writing – review and editing. **Karan Chaman Lal:** writing – original draft, writing – review and editing. **Zahabia Adnan:** visualization, writing – original draft, writing – review and editing. **Shamyl Zehra:** writing – original draft, writing – review and editing. **MD. Faisal Ahmed:** writing – review and editing.

## Funding

The authors have nothing to report.

## Ethics Statement

The authors have nothing to report.

## Consent

Written informed consent was obtained from the patient's legal guardian for publication of this case report.

## Conflicts of Interest

The authors declare no conflicts of interest.

## Data Availability

Not publicly available due to privacy and ethical restrictions.
